# Novel Methods for Prevention of Hydrogen Embrittlement in Iron

**DOI:** 10.1038/s41598-017-17263-8

**Published:** 2017-12-05

**Authors:** Q. Xu, J. Zhang

**Affiliations:** Research Reactor Institute, Kyoto University, Osaka, 590-0494 Japan

## Abstract

Iron is the most widely used metal in the world. However, hydrogen embrittlement in steels—iron based alloys—is an important issue related to the safety of our infrastructure, such as railroads and bridges. Therefore, the prevention of hydrogen embrittlement in steels is necessary. In the present study, we demonstrate two novel methods for the prevention of hydrogen embrittlement in iron: one involves the low-energy implantation of helium, which is usually an element harmful to metals, into iron, the other is inducing damage to the iron surface by ion irradiation. In general, irradiation with high-energy particles leads to metal brittleness. In the former method, the driving force for hydrogen embrittlement in iron is weakened, in the latter method, hydrogen diffusion in iron is prevented because of trapping of hydrogen atoms in the vacancies produced by the irradiation. As a result, hydrogen embrittlement in iron was suppressed by both methods.

## Introduction

Hydrogen, which is the most abundant element present the universe, is known to degrade the performance of structural materials; this phenomenon is generally called hydrogen embrittlement. Iron and its alloys, i.e., steels, are widely used as structural materials in infrastructure, such as railways, roads, and bridges. The investigation of hydrogen embrittlement of steels has been performed for over 140 years. However, the mechanism of hydrogen embrittlement of steels is not yet clear. Thus far, two mechanisms for hydrogen embrittlement of metals have been presented^1^. One is called hydrogen-enhanced localized plasticity (HELP)^2–4^, and the other is hydrogen-induced decohesion (HID)^5–7^. The former is based on experimental results that demonstrate that hydrogen enhances the mobility of dislocations by decreasing the strength of the barriers to dislocation motion. The latter is based on numerical calculation results that indicate that hydrogen reduces the cohesive strength of a solid along crystallographic planes, particle/matrix interfaces, or grain boundaries, and then decreases the fracture toughness of the materials.

Two strategies for reducing and preventing hydrogen embrittlement in steels have been developed. The first strategy is to reduce the intake of hydrogen by applying coating that act as diffusion barriers to prevent the ingress of hydrogen. The second strategy is to render the hydrogen innocuous by reducing the mobility of hydrogen atoms in the steel, typically by hydrogen trapping^8^.

Recently, it was reported that helium trapped by dislocations in iron and nickel increases the total elongation of these metals^9–11^. These experimental results were contrary to expectation. It is typically reported that diffusive helium adversely affects the toughness of metals. This is because helium atoms are trapped by the vacancy clusters induced by neutron irradiation, and these form helium bubbles in the matrix and grain boundaries, causing embrittlement. However, it has been reported that if the helium atoms are trapped by dislocations rather than vacancies, then the total elongation increases. The reason why dislocation trapping of helium increases elongation is not yet clear. However, it has been suggested that helium atoms trapping by dislocations desorb during dislocation motion, and these atoms are trapped by vacancies induced by tensile testing. This makes it difficult for vacancies to aggregate into voids, leading to the increase in elongation^10^. Although damage induced by irradiation with high-energy particles usually leads to embrittlement, it is well known that the vacancies induced by irradiation can trap hydrogen atoms, and this can lead to a decrease in the hydrogen diffusion rate. Therefore, in the present study, the effects of helium atom trapping at dislocations and irradiation-induced vacancy-clusters on the hydrogen embrittlement have been investigated.

## Results

### Effect of hydrogen on mechanical properties of annealed sample

**Figure 1:**
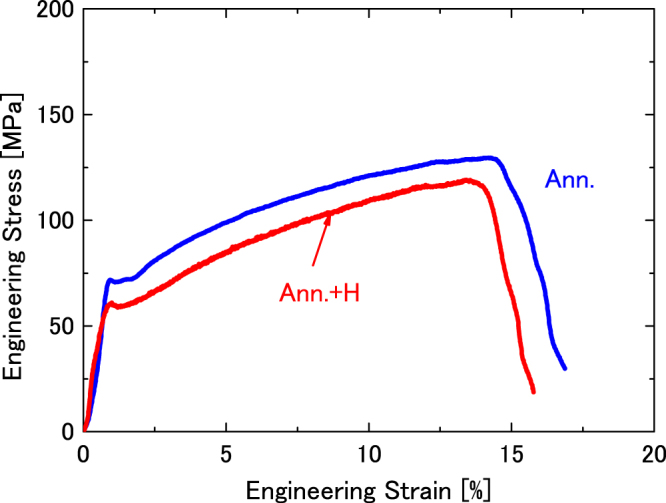
Engineering stress– engineering strain curves for annealed (Supplementary Fig. 1, Procedure I), and H^+^–implanted samples (Supplementary Fig. 1, Procedure II).

Figure 1 shows engineering stress– engineering strain curves for an annealed sample (Supplementary Fig. 1, Procedure I) and after H^+^ implantation (Supplementary Fig. 1, Procedure II). The mechanical properties of yield stress, tensile strength, uniform elongation, and total elongation for all experiments are summarized in Table 1. As shown in Fig. 1, the two tensile stress–strain curves are almost parallel, but the values of stress in the stress–strain curve of H^+^–implanted sample are low. The yield stress and tensile strength of the H^+^–implanted sample were lower than that of the well-annealed sample, and decreased from 71 to 60 MPa, and from 129 to 119 MPa, respectively. Simultaneously, the total elongation decreased from 14.4% to 12.6% as a result of H^+^ implantation. The elongation in both samples was mainly uniform elongation.

**Table 1:** Typical mechanical properties of samples after various experimental procedures.

Experimental procedure of sample (Supplementary Fig. 1)	Yield stress (MPa)	Tensile strength (MPa)	Uniform elongation (%)	Total elongation (%)
Procedure I	71 ± 5	129 ± 12	14.1 ± 0.5	14.4 ± 1.2
Procedure II	60 ± 4	119 ± 10	12.1 ± 0.9	12.6 ± 1.1
Procedure III	163 ± 4	175 ± 8	6.3 ± 0.3	8.7 ± 0.9
Procedure IV	156 ± 5	162 ± 8	2.3 ± 0.3	3.6 ± 0.8
Procedure V	161 ± 3	168 ± 9	6.8 ± 0.5	7.5 ± 1.0
Procedure VI	153 ± 6	159 ± 10	5.6 ± 0.6	6.4 ± 1.2
Procedure VII^a^	152 ± 5	160 ± 7	1.3 ± 0.3	4.0 ± 0.7
Procedure VII^b^	144 ± 3	161 ± 11	6.8 ± 0.4	8.0 ± 0.8

^a^The Fe irradiation dose was 0.075 dpa (displacement per atom); ^b^The Fe irradiation dose was 0.75 dpa.

### Effect of He^+^ implantation on hydrogen embrittlement in annealed cold-rolled sample

**Figure 2:**
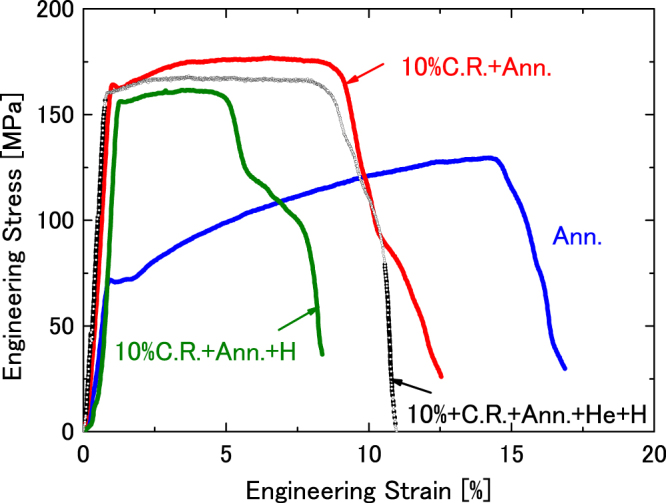
Engineering stress– engineering strain curves for annealed cold-rolled (Supplementary Fig. 1, Procedure III), and H^+^–implanted samples without (Supplementary Fig. 1, Procedure IV) and with He^+^–implantation (Supplementary Fig. 1, Procedure V). A similar curve for the annealed sample (Supplementary Fig. 1, Procedure I) was also added for comparison.

Figure 2 shows engineering stress– engineering strain curves for an annealed sample after deformation to 10% (Supplementary Fig. 1, Procedure III), and the same samples after H^+^ implantation without (Supplementary Fig. 1, Procedure IV) and with He^+^ implantation (Supplementary Fig. 1, Procedure V). A similar curve for a well-annealed sample (Supplementary Fig. 1, Procedure I) is also plotted for comparison. Compared with the well-annealed sample, in the annealed cold-rolled sample, the yield stress and tensile strength increased (from 71 to 163 MPa and from 129 to 175 MPa, respectively) and the total elongation decreased (from 14.4% to 8.7%). H^+^ implantation slightly decreased the yield stress and tensile strength, but markedly decreased the total elongation of the annealed cold-rolled sample. The yield stress, tensile strength, and total elongation decreased from 163 to 156 MPa, from 175 to 162 MPa, and from 8.7% to 3.6%, respectively. He^+^ implantation before H^+^ implantation into the annealed cold-rolled sample to result in decreased reduction in the total elongation. As detailed in Table 1, the changes in the yield stress and tensile strength were small; however, the total elongation increased from 3.6% to 7.5%. Compared with the well-annealed sample, the proportion of uniform elongation decreased in the annealed cold-rolled sample, and it decreased further after H^+^ implantation. Conversely, He^+^ implantation increased the proportion of uniform elongation.

### Effect of surface damage on hydrogen embrittlement in annealed cold-rolled sample

**Figure 3:**
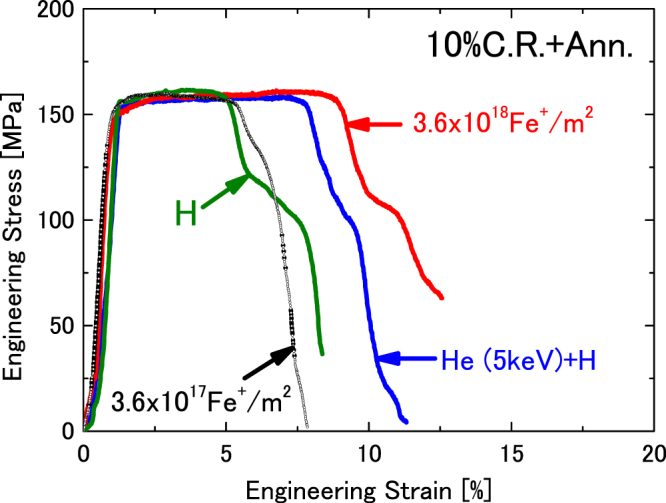
Engineering stress– engineering strain curves for H^+^–implanted annealed cold-rolled sample (Supplementary Fig. 1, Procedure III), and the curves for samples that were pre-irradiated by He^+^ (Supplementary Fig. 1, Procedure VI) and Fe^+^ ions (Supplementary Fig. 1, Procedure VII). Two kinds of Fe^+^ ion irradiation doses were used.

The effects of irradiation with 5 keV He (Supplementary Fig. 1, Procedure VI) or 1 MeV Fe (Supplementary Fig. 1, Procedure VII) ions on the hydrogen embrittlement in iron are shown in Fig. 3. Since there is no sharp yield point in the nominal tensile stress–strain curves of this sample set, the yield stress of the ion irradiated samples is determined by using the 0.2% offset yield stress method (Table 1). The tensile strengths of the H^+^–implanted annealed cold-rolled sample and the sample irradiated with He or Fe ions before H^+^ implantation did not vary, but the reduction in total elongation was improved from 3.6% to 6.4% and 8.0% for the cases of He^+^ irradiation and Fe^+^ irradiation before H^+^ implantation, respectively. Although the damage doses in both Fe (0.73 dpa) and He (0.8 dpa) ion irradiation were very similar, the total elongation of the He^+^–irradiated sample (6.4%) was lower than that of the Fe^+^ irradiated sample (8.0%). This improvement depended strongly on the of ion irradiation dose. With increasing Fe ion irradiation dose from 3.6 × 10^17^ to 3.6 × 10^18^ Fe^+^/m^2^, the total elongation increased from 4.0% to 8.0%. In addition, the proportion of uniform elongation increased with increasing damage dose.

**Figure 4:**
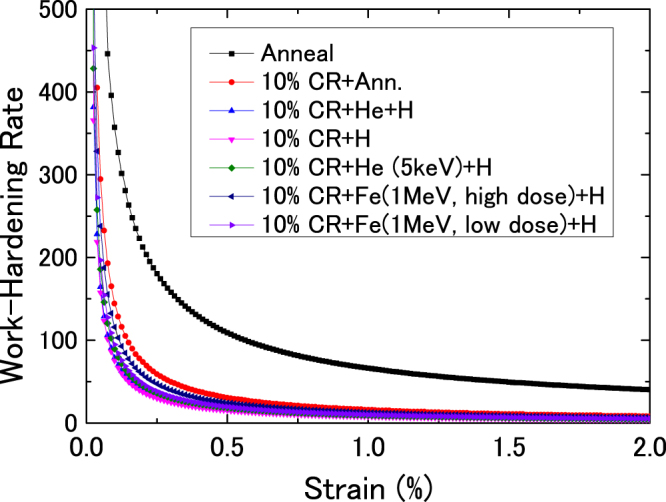
Relation of work hardening rate and true strain in well annealed, annealed cold-rolled samples, and annealed cold-rolled samples after He^+^ and H^+^, H^+^ implantations, and H^+^–implanted annealed cold–rolled samples with pre-damage induced by 5 keV-He, and 1 MeV-Fe ions, respectively. Two kinds of Fe^+^ ion irradiation doses were used.

Figure 4 shows the relation of work hardening rate and true strain in He^+^– and H^+^–, and H^+^–implanted annealed cold-rolled samples; and these samples with pre-damaged by ion irradiation. For comparison, the relation of the work hardening rate of the true strain in a well-annealed sample is also shown. In all cases, the work hardening rate decreased with increasing strain in the initial stage, and then it saturated with increasing strain. The well-annealed sample has the highest work hardening rate, followed by the annealed cold-rolled sample. Although the changes in the work hardening rate against true strain in He^+^– and H^+^–, and H^+^–implanted annealed cold-rolled samples and these samples with pre-damaged by irradiation were small, the annealed cold-rolled sample after H^+^ implantation has the lowest work hardening rate.

### Microstructures near the fracture

**Figure 5:**
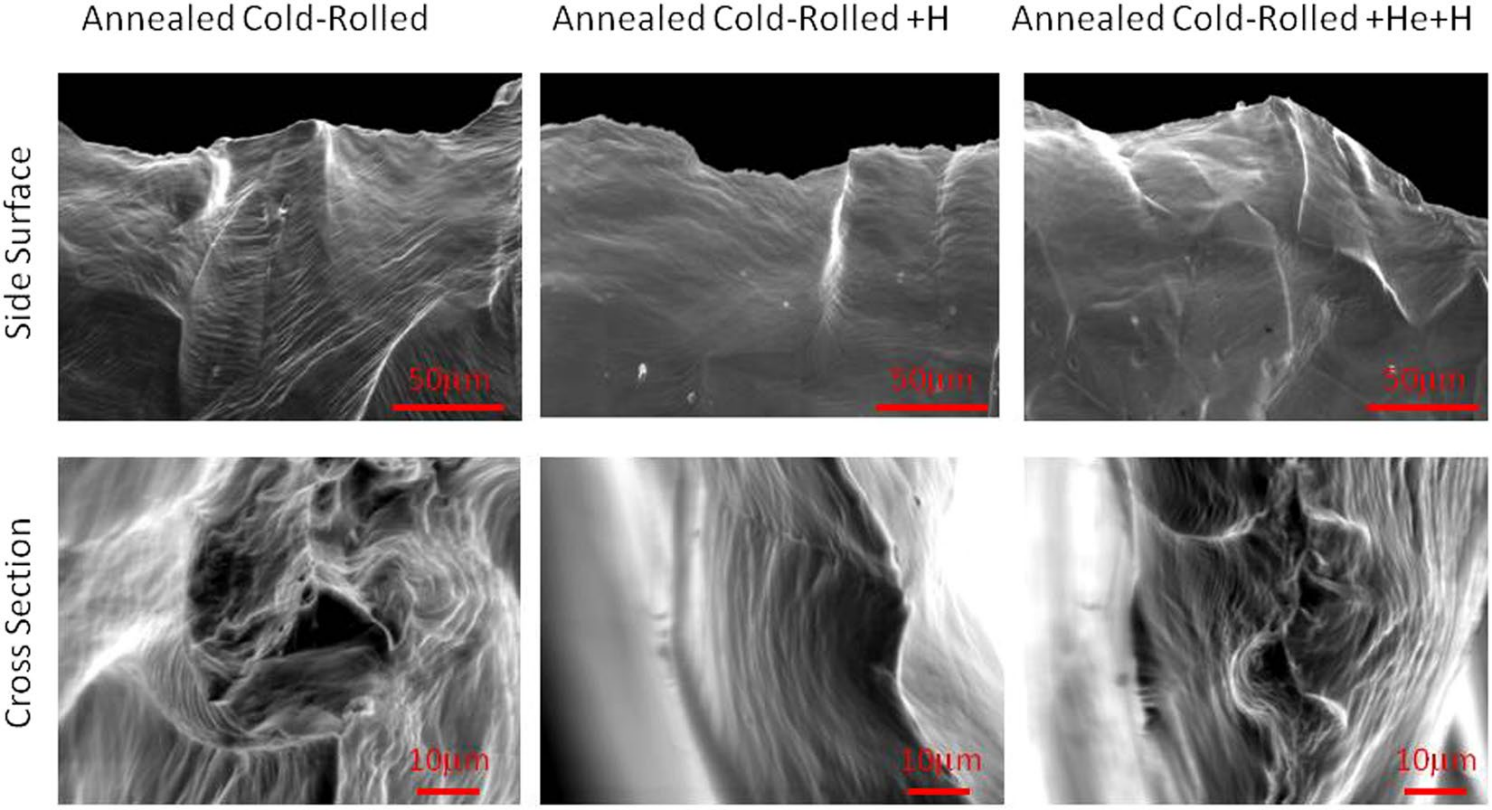
Fracture surface images of annealed cold–rolled sample (left), and these samples after H^+^ (middle), He^+^ and H^+^ implantations (right). The upper part is the side surface of fracture, and the lower part is the cross section of fracture.

Figure 5 shows the fracture surface images of annealed cold-rolled and these samples after single implantation of H, and combined implantation of He and H. The upper part of the figure is the side surface of fracture, and the lower part of the figure is the cross section of fracture. As shown in the figure, the fracture mode is ductile, and no significant difference in the fracture was observed. In order to investigate the microstructural evolution during the tensile test in annealed cold-rolled samples after He^+^ and H^+^ implantations, positron lifetime measurements were performed. Table 2 shows the results of positron lifetime measurements. Here, τ_m_ is the positron mean lifetime obtained from the single component analysis of the measured lifetime spectra. Compared with the well-annealed sample lifetime of 106.3 ps^12^, the long lifetime component, τ_2_, near the fracture of the annealed cold-rolled sample was 209.3 ps with an intensity of 45.8%, which corresponds to a vacancy cluster with two vacancies. Here, τ_2_ decreased to 166.9 ps (lower than positron lifetime of a single vacancy (180 ps^13^)) in the annealed cold-rolled sample after He^+^ implantation. However, τ_2_ were 181.9 and 169.6 ps in the annealed cold-rolled sample after single implantation of H, and combined implantation of He and H, respectively.

**Table 2:** Results of positron lifetime for several samples after tensile testing.

	Procedure III	Procedure III+He	Procedure IV	Procedure V
Mean Lifetime (τ_m_, ps)	132.6 ± 0.2	141.0 ± 0.3	146.8 ± 0.3	148.1 ± 0.3
Short Lifetime (τ_1_, ps)	93.4 ± 1.6	107.0 ± 7.0	100.0 ± 4.4	78.6 ± 7.4
Long Lifetime (τ_2_, ps)	209.3 ± 10.5	166.9 ± 7.4	181.9 ± 5.5	169.6 ± 2.3
Intensity (I_2_, %)	45.8 ± 4.4	51.4 ± 11.3	51.6 ± 5.3	78.1 ± 4.3

## Discussion

Generally, two methods are employed to reduce hydrogen embrittlement in iron. One involves weakening the interactions between hydrogen and iron, and the other is based on preventing hydrogen from entering the iron sample. Tian *et al*. reported that palladium reduces the embrittlement energy of hydrogen on the iron grain boundary by −0.1 eV, and thus improves hydrogen embrittlement resistance^14^. As described in the introduction section, the coating and hydrogen trapping methods are effective ways to prevent the entry of hydrogen into iron. Hydrogen atoms can easily migrate through a perfect α-iron lattice owing to its small migration energy of 0.14 eV^15^. Hydrogen atoms are insoluble in iron at room temperature. It is favorable for the hydrogen to be trapped by sinks, such as vacancy clusters, interstitial clusters, dislocations, and grain boundaries. Therefore, hydrogen is not present if there are no defects in iron. As shown in Fig. 1, H^+^–, even when implanted in well-annealed iron, decreased the yield stress, tensile strength, and total elongation owing to the presence of dislocations in iron. Even in well-annealed metals, dislocations with a density of 10^10^–10^12^ m/m^3^ are present. Implanted hydrogen atoms were trapped by dislocations in the well-annealed iron, and this degraded the mechanical properties of the specimens subject to H^+^ implantation.

**Figure 6:**
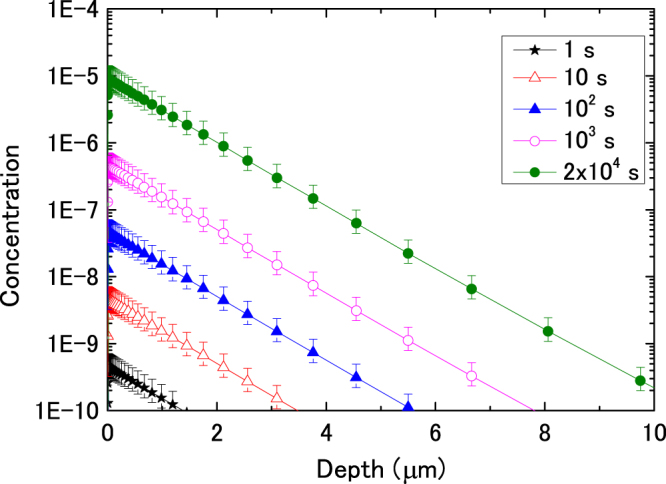
Depth and time dependence of hydrogen concentration during implantation at an energy of 100 eV and a flux of 1.0 × 10^16^ H^+^/m^2^s in annealed cold–rolled sample.

Similar to the analysis of helium diffusion in annealed cold-rolled iron^9^, the hydrogen diffusion behavior was simulated in the present study. In the calculations, the distribution of implanted hydrogen, which had a rectangular shape, was not the same as that shown in Supplementary Fig. 2. The center of the rectangular shape was 5 nm from the incident surface, and its width was also 5 nm. The migration energy of hydrogen atoms was 0.14 eV. The hydrogen concentration at both surfaces was always zero. The rate of depth change in the hydrogen concentration can be expressed as^9^

1 $$\frac{{{\rm{dC}}}_{{\rm{H}}}}{{\rm{dt}}}={{\rm{P}}}_{{\rm{H}}}({\rm{x}})+{{\rm{D}}}_{{\rm{H}}}\frac{{\partial }^{2}{{\rm{C}}}_{{\rm{H}}}}{\partial {{\rm{x}}}^{2}}-\frac{{{\rm{D}}}_{{\rm{H}}}}{{{\rm{a}}}^{2}}{{\rm{C}}}_{{\rm{H}}}({{\rm{Z}}}_{{\rm{D}}-{\rm{H}}}{{\rm{C}}}_{{\rm{D}}}+{{\rm{Z}}}_{{\rm{S}}-{\rm{H}}}{{\rm{C}}}_{{\rm{S}}})$$

2 $$\frac{{{\rm{dC}}}_{{\rm{HD}}}}{{\rm{dt}}}=\frac{{{\rm{D}}}_{{\rm{H}}}}{{{\rm{a}}}^{2}}{{\rm{C}}}_{{\rm{H}}}{{\rm{Z}}}_{{\rm{D}}-{\rm{H}}}{{\rm{C}}}_{{\rm{D}}}$$

where *P*_H_ is the implantation rate of hydrogen, *P*_H_ is 0 at depths greater than 7.5 nm, and *D*_H_ is the diffusion coefficient of hydrogen. *a* is one atomic distance. *Z* is the site number of the reaction. Here, we assume *Z* to be 1 in all reactions. The rate of depth change in the hydrogen concentration as shown in equation (1) is equal to the difference between the increase, which includes implanted hydrogen (the first term on the right–hand side of equation (1)) and diffusion of hydrogen (the second term on the right–hand side of equation (1)), and decrease, which is hydrogen trapped by dislocations and other sinks, such as the surface and grain boundaries (the third term on the right–hand side of equation (1)), in the amount of mobile hydrogen. Positive and negative terms indicate increases and decreases in the amount of mobile hydrogen, respectively. Equation (2) is the rate of depth change in the concentration of hydrogen trapped by dislocations. The parameters employed in these calculations are shown in Table 3. *D*_H_ was evaluated to be 2.8 × 10^9^ nm^2^/s according to ref^11^. The calculation results are shown in Fig. 6. After H^+^ implantation (2 × 10^4^ s), the concentration of hydrogen trapped by dislocations reached 1 × 10^−6^ within 3 μm of the surface in annealed cold-rolled iron. This means that almost all hydrogen trapping sites, i.e., dislocations trapped hydrogen atoms. The hydrogen concentration decreased gradually with increasing depth. The depth of several micrometers induced by diffusion of hydrogen was much wider than the hydrogen implantation range estimated using SRIM as shown in Supplementary Fig. 2. Therefore, the diffusion of hydrogen deeper into the matrix affected the mechanical properties of iron samples containing only dislocations. In addition, the total amount of hydrogen trapped by the dislocations was estimated to be about 10^18^ H/m^2^ from Fig. 6, which is lower than the implantation dose of hydrogen. Most of the hydrogen escaped from the surface of the sample.

**Table 3:** Typical parameters used in calculations.

*P*_H_ (s^−1^)	1.2 × 10^−5^
*D*_H_ (nm^2^/s)	2.8 × 10^9^
*C*_D_	1.2 × 10^−6^ ^9^
*C*_S_	10^−10^
*Z*	1
*a*	0.249 nm
*T* (K)	300

As shown in Figs 1 and 2, the yield point phenomenon appeared in well-annealed and annealed cold-rolled samples. This phenomenon is associated with interstitial impurities, such as carbon and nitrogen, as listed in Supplementary Table 1, which are pinning the existing dislocations. H^+^ implantation decreased the yield point in the annealed cold-rolled sample, as shown in Fig. 2. There are two interpretations of the yield point reduction. First, hydrogen attaches itself to dislocations that are freed from carbon or nitrogen by thermal fluctuations^16^. Next, hydrogen enhances the local plasticity (HELP) mechanism and change in the distribution of the dislocation^17^. Although the yield point did not appear significantly in the annealed cold-rolled sample after He^+^ and H^+^ implantations, the yield stress also decreased (dark curve); however, it was higher than the yield stress in the annealed cold-rolled sample after H^+^ implantation (green curve). As indicated by Xu *et al*., similar to hydrogen, the interstitial helium decreased the yield point in iron^9^. If the decrease in yield stress is due to the former mechanism, the decreased in yield stress in the sample after He^+^ and H^+^ implantations is more pronounced. Therefore, the decrease in yield stress in the present study is attributed to the latter mechanism (HELP). In addition, in the present study, the amount of impurities, such as carbon and nitrogen, has not reached a level that will influence of the amount of hydrogen trapped by the dislocations.

Dislocations induced by cold work increase the yield stress and tensile strength, and decrease the total elongation, as shown in Fig. 2 (red curve). This phenomenon is called as work hardening. Hydrogen trapped at dislocations decreased the total elongation from 8.7% to 3.6%, as shown by the green curve of Fig. 2. The yield stress and tensile strength were also slightly reduced. Compared with the H^+^–implanted well-annealed sample, the annealed cold-rolled sample demonstrated a more prominent reduction in the total elongation induced by hydrogen. This is because the dislocation density in the annealed cold-rolled sample is higher than that in the well-annealed sample, thus the amount of hydrogen trapped in dislocations is also higher in that case. However, implanted helium reduced the extent of hydrogen embrittlement, as shown by the black curve of Fig. 2. The total elongation improved from 3.6% to 7.5%. The yield stress and tensile strength also increased slightly. Unexpectedly, it was reported that helium trapped by the dislocations improves the ductility of iron and nickel^9–11^. Although the mechanism for improving the ductility of iron and nickel by helium is still not clear, it is suggested that helium delays the aggregation of vacancies produced during sample tension, resulting in an increase in elongation. It was impossible to obtain the microstructures by measuring the positron lifetime very near the fracture in the present study, because the size of the positron source was 2 mm in diameter. Table 2 shows the positron lifetimes in the vicinity of fracture with the width of about 2 mm. It is clear that helium suppresses the aggregation of vacancies during tensile testing in the He^+^–implanted annealed cold-rolled sample since the long lifetime component, τ_2_, decreased from 209.3 to 166.9 ps. As shown in Table 2, the long lifetime component, τ_2_, in the H^+^–implanted annealed cold-rolled sample (Supplementary Fig. 1, Procedure IV) was lower than that in the annealed cold-rolled sample (Supplementary Fig. 1, Procedure III); this indicates that the tensile sample broke before vacancy aggregation occurred in the H^+^ implanted annealed cold-rolled sample. Hydrogen weakened the interaction between iron atoms. Helium also suppressed the aggregation of vacancies in the H^+^–implanted annealed cold-rolled sample, since the long lifetime component, τ_2_, in the He^+^– and H^+^–implanted annealed cold-rolled sample (Supplementary Fig. 1, Procedure V) was lower than that in H^+^–implanted annealed cold-rolled sample (Supplementary Fig. 1, Procedure IV).

Both helium and hydrogen are produced by nuclear reactions: (n, α) and (n, p). Recently, Erhart and Marian reported that only about 3% of helium atoms were trapped by the cascade produced by the nuclear reaction of (n, α) in iron^18^, and most of the helium atoms could diffuse in the long-term. Therefore, determining the behavior of helium and hydrogen is important in the nuclear industry, and this need increases with advances in fusion reactor technology. It has been indicated that, when both helium and hydrogen are present, the void growth is more significant compared with that when only helium or hydrogen are present^19–22^. In addition, attractive interaction has been reported between substitutional He and interstitial H in the iron matrix^23^. However, it was reported recently that the interaction between helium and hydrogen inside a void is weak, and this conclusion is based on both experimental results^24^ and atomistic simulations^25^. Helium atoms occupy the center of the bubble, while the hydrogen atoms occupy the near surface of the bubble. This means that the core of the bubble is composed of helium, surrounded by a shell of hydrogen atoms. Moreover, this configuration does not depend on the bubble size. Hydrogen atoms prefer to bond to the iron atoms, but the interaction of hydrogen and helium is weak. However, it is possible that the H–Fe interaction deteriorates as the He–H interaction increases at the dislocation core, since the distance between the helium atom and the hydrogen atom in the dislocation is shorter than that in a vacancy. Although the present results indicate that helium delays the aggregation of vacancies produced during sample tension as described above, it is necessary to verify whether helium influences the interaction between hydrogen and iron using ab initio calculations in the future.

The interactions between hydrogen and vacancies and between hydrogen and dislocations in α-iron have been investigated^24,26–28^. Dislocations and vacancies trap the hydrogen and reduce the hydrogen diffusion in iron. However, the presence of hydrogen traps increases the saturation hydrogen content in iron. The hydrogen de–trapped from trapping sites will enhance the hydrogen embrittlement in iron. As described above, the hydrogen trapped by the dislocations decreased the yield stress, tensile strength, and the total elongation, as shown in Fig. 2. Compared with engineering stress– engineering strain curves of annealed samples with and without H^+^ implantation, degradation of mechanical properties due to the hydrogen is significant in the cold-rolled sample, where the dislocation density is high. Therefore, it is impossible to reduce or prevent hydrogen embrittlement due to dislocations in iron. However, as shown in Fig. 3, the defects induced by the ion irradiation improved the hydrogen embrittlement, and this tendency became stronger as the irradiation dose increased. Voids (vacancy cluster) and interstitial type dislocation loops (interstitial cluster) are formed by the ion irradiation. Tynan *et al*. also indicated that pre-damaged tungsten increased deuterium retention^29^. Since the dislocations cannot reduce the hydrogen embrittlement in iron, the formation of voids during ion irradiation is a key point to improve the hydrogen embrittlement in the present study. The voids trap the hydrogen and delay the hydrogen diffusion in iron. Although the He irradiation dose was slightly higher than the Fe dose, Fe ion irradiation is more effective in suppressing hydrogen embrittlement. This is because the damage region formed by Fe ion irradiation is wider than that produced by the He ions.

Although the elongation decreased in the H^+^ implanted samples in the present study, the ductile fracture could not be observed in those samples, as shown in Fig. 5. In addition, hydrogen decreased the uniform elongation. As shown in Table 1, the proportion of uniform elongation decreased with an increase in the amount of hydrogen in the matrix of sample.

## Conclusion

Although the mechanism of hydrogen embrittlement in steels is not clear, it is important to prevent or reduce this phenomenon since it threatens the safety of the structural materials used in infrastructure. Based on the experimental results of this study, two novel methods for the prevention of hydrogen embrittlement in iron are presented. Firstly, helium atoms (which usually adversely affect metal properties) trapped in dislocations to reduce the hydrogen embrittlement in iron. Secondly, the formation of vacancy clusters induced by ion irradiation near the iron surface traps hydrogen and delays the hydrogen diffusion, thereby, suppressing hydrogen embrittlement. The hydrogen embrittlement suppression improves with an increase in the ion irradiation dose.

## Methods

Rod-shaped polycrystalline pure iron specimens (99.995%, Johnson–Matthey) were used. The primary impurities in iron are listed in Supplementary Table 1. The experimental procedures employed for specimen preparation are shown in Supplementary Fig. 1. Sheets, which were obtained by rolling the iron rods, were annealed at 973 K for 2 h in a hydrogen atmosphere (Procedure I). Defects were produced by rolling the well-annealed specimens to 90% of the original thickness. In addition, dislocations, vacancies and vacancy clusters were also produced in the specimens. Tensile samples of 0.1 mm in thickness were punched from the rolled sheets^9^. According to previous experimental results^9^, the vacancies and vacancy clusters are removed and only dislocations remain after the 10% elongated tensile samples were annealed at 673 K for 1 h under a vacuum of pressure lower than 1 × 10^−4^ Pa (Procedures III to VII). This heat treatment was performed immediately after cold rolling. The dislocation density decreased after annealing. The average grain size of the annealed sample (Procedure I) was 32 μm; the average grain size, which was 39 μm, became slightly larger after cold rolling. The average grain size, which was 40 μm, only experienced a minimal change during annealing at 673 K for 1 h (Procedure III). H^+^ implantation was used to introduce hydrogen into specimens. H^+^ implantation was performed on tensile samples at room temperature in a vacuum chamber using an ion gun, in which the energy of ions was controlled strictly. The energy of H^+^ implantation was 350 eV, which cannot damage iron under the assumption of threshold displacement energy of 24 eV^30^. The H^+^ dose was 2.0 × 10^20^ H^+^/m^2^ at a flux of 1.0 × 10^16^ H^+^/m^2^s (Procedures II, and IV to VII). The H distribution in iron as shown in Supplementary Fig. 2 was estimated using the SRIM code^31^. The implanted H ions were deposited at 0–15 nm from the incident surface, and the maximum H distribution was ~5 nm from the surface. The introduction of helium was also performed using the same gun for Procedure V. The energy of He^+^ implantation was selected to be 100 eV to avoid displacement damage. The nominal He^+^ dose was 1.0 × 10^20^ He^+^/m^2^ at a flux of 5.0 × 10^15^ He^+^/m^2^s (Procedure V). As shown in Supplementary Fig. 2, the implanted He ions were deposited at 0–4 nm from the incident surface and the peak of He distribution was ~1 nm from the incident surface. In order to reduce the diffusion of hydrogen from the surface of the sample, irradiation with two different ions and different damage ranges were performed (Procedures VI and VII). One was the He^+^ irradiation using an ion gun, and the other was Fe^+^ irradiation using the tandem pelletron accelerator in the Quantum Science and Engineering Center, Kyoto University^32^. The energy of He^+^ irradiation was 5 keV, and the dose was 1.0 × 10^20^ He^+^/m^2^. The energy of Fe^+^ irradiation was 1 MeV, the doses were 3.6 × 10^17^ and 3.6 × 10^18^ Fe^+^/m^2^. Similar to the distribution of ion deposition, as shown in Supplementary Fig. 2, the damage induced by the 5 keV He ions and 1 MeV Fe ions also depends on the ion penetration depth. According to the calculation of the SRIM code, the damage ranges induced by the He and Fe ions were 0–60 and 0–600 nm from the incident surface and the maximum damage was ~25 and ~280 nm from the incident surface, respectively (Supplementary Fig. 3). The damage peaks induced by the He and Fe irradiation were 0.8 and 0.075, 0.75 dpa (displacement per atom), respectively. Fe^+^ as well as He^+^ irradiations, and He^+^ as well as H^+^ implantations did not change the grain size and dislocation density of iron.

The tensile tests were performed at 300 K with a strain rate of 2 × 10^−3^/s. Three experiments were conducted under the same conditions. Strain and elongation were estimated by measuring the distance moved by the cross-head. Although deformation of the gauge section induced by high tensile force lead to overvaluation of the real strain, the tensile force used in the present study was too low to deform the gauge section. In addition, the displacement of the cross-head was calibrated before the tensile tests.

The formation of defect clusters, especially vacancy-type defects, after the tensile test was investigated by positron annihilation spectroscopy (PAS). Here ^22^Na with a diameter of 2 mm was used as a positron source. The time resolution of the positron lifetime spectrometer was 190 ps (full–width at half–maximum, FWHM), and the total count for each spectrum was over 1 × 10^6^. Based on the two-state trapping model, the lifetime spectrum *L(t)* could be divided into two components: short lifetime, τ_1_, and long lifetime, τ_2_. The former component corresponds to the bulk, and the latter component corresponds to defects. The lifetime spectrum can be expressed as follows:^33^

3 $$L(t)=({I}_{1}/{\tau }_{1})exp(-t/{\tau }_{1})+({I}_{2}/{\tau }_{2})exp(-t/{\tau }_{2})$$

where *I*_*i*_ are the intensities of lifetimes (*I*_*1*_ + *I*_*2*_ = 1). The lifetimes, *τ*_1_ and *τ*_2_, and mean positron lifetime, *τ*_*m*_, are written as follows:

4 $${t}_{{1}}={1}/{{\lambda }}_{{1}}={1}/({\lambda }_{B}+k),$$

5 $${t}_{{2}}={1}/{\lambda }_{{2}}={1}/{{\lambda }}_{d},$$

6 $$k={I}_{{2}}({{\lambda }}_{{1}}-{{\lambda }}_{{2}}),$$

7 $${t}_{m}={I}_{1}{\tau }_{1}+{I}_{2}{\tau }_{2},$$

where *k* is the defect trapping rate, and *λ*_*B*_ and *λ*_2_ are the annihilation rates in the bulk and at defects, respectively. As shown in equation (4), the bulk lifetime in the matrix without defects is *τ*_*B*_ = *τ*_*m*_ = *1*/*λ*_*1*_ = *1*/*λ*_*B*_. Therefore, the short lifetime, τ_1_, is shorter than the bulk lifetime, *τ*_*m*_, in the matrix containing only one type of defect.

## Electronic supplementary material

Supplementary Information
