# Supplementary material for: Novel Methods for Prevention of Hydrogen Embrittlement in Iron
Source: Sci Rep. 2017 Dec 5;7:16927. doi: 10.1038/s41598-017-17263-8 (PMC5717234; doi:10.1038/s41598-017-17263-8)
Supplement: Supplementary file 1 — Supplementary Information [file 41598_2017_17263_MOESM1_ESM.pdf]

Supplementary Materials

Novel Methods for Prevention of Hydrogen Embrittlement in Iron

Q. Xu[*], *J. Zhang*

Research Reactor Institute, Kyoto University, Osaka 590-0494, Japan

[*] Corresponding author, Q. Xu; Email: xu@rri.kyoto-u.ac.jp.

Supplementary Table 1   Primary impurities in pure iron (in wt ppm)

C	N	O	P	Cu	Mn	Co	Cr	Ni	Al
44	<10	34	3.9	2.3	2.7	2.5	2.7	1.3	5.5

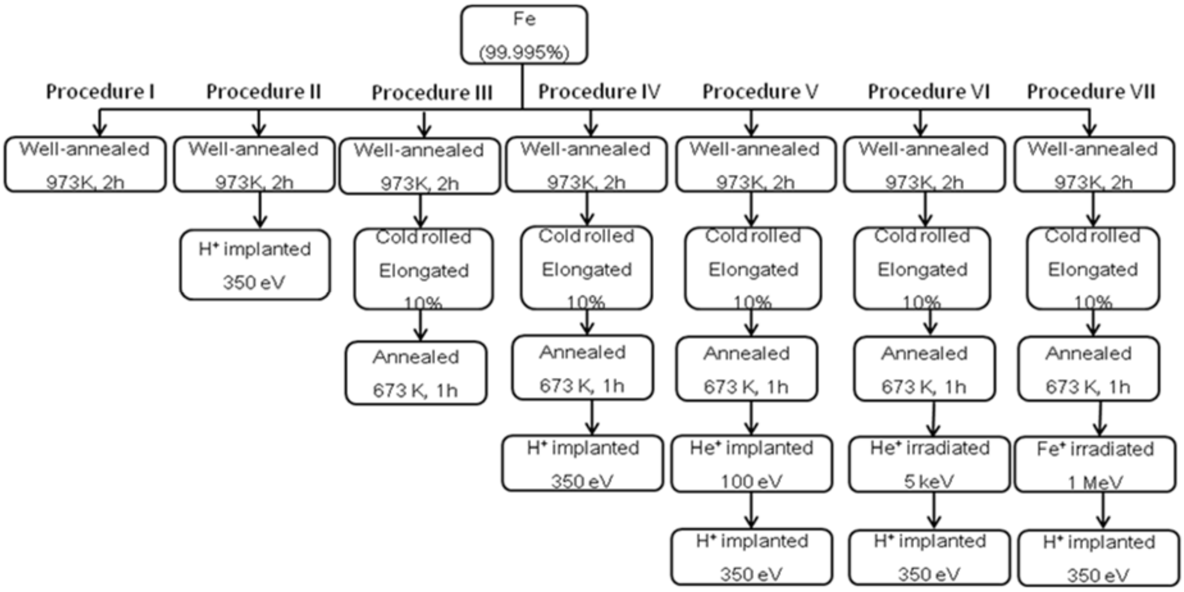

Supplementary Figure 1. Preparation of specimens.

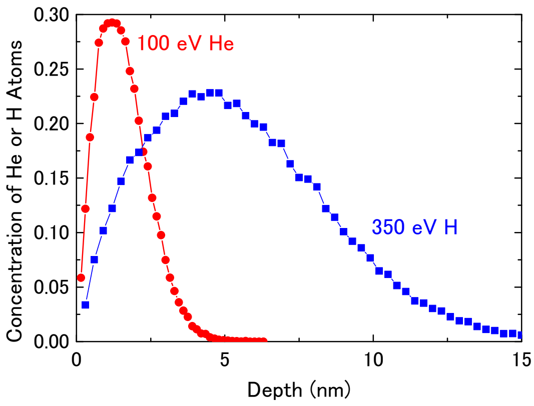

Supplementary Figure 2. Depth distribution of ions in iron implanted with He ions at 100 eV and H ions at 350 eV with doses up to 1×10^{20} and 2×10^{20} ions/m^2, respectively. No displacement damage was produced by 100 eV He ions and 350 eV H ions.

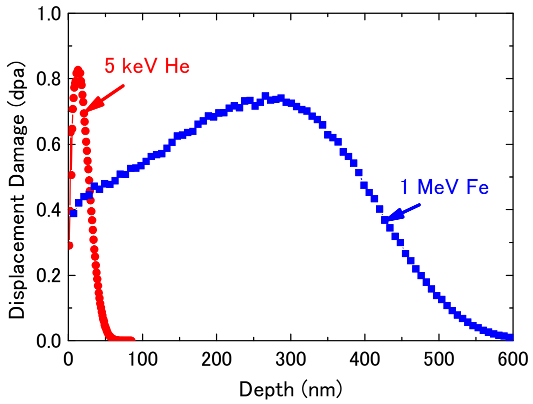

Supplementary Figure 3. Depth distribution of damages in iron irradiated with 5 keV He at a dose up to 1×10^{20} ions/m^2 and with 1 MeV Fe ions at a dose up to 3.6×10^{18} ions/m^2, respectively.
